# A Metabolomic Signature of Obesity and Risk of Colorectal Cancer: Two Nested Case–Control Studies

**DOI:** 10.3390/metabo13020234

**Published:** 2023-02-05

**Authors:** Mingjia Yang, Chen Zhu, Lingbin Du, Jianv Huang, Jiayi Lu, Jing Yang, Ye Tong, Meng Zhu, Ci Song, Chong Shen, Juncheng Dai, Xiangfeng Lu, Zekuan Xu, Ni Li, Hongxia Ma, Zhibin Hu, Dongfeng Gu, Guangfu Jin, Dong Hang, Hongbing Shen

**Affiliations:** 1 Department of Epidemiology, School of Public Health, Southeast University, Nanjing 210009, China; 2 Department of Epidemiology, Center for Global Health, School of Public Health, Nanjing Medical University, 101 Longmian Avenue, Nanjing 211166, China; 3 Department of Cancer Prevention, The Cancer Hospital of the University of Chinese Academy of Sciences (Zhejiang Cancer Hospital), Hangzhou 310022, China; 4 Institute of Basic Medicine and Cancer (IBMC), Chinese Academy of Sciences, Hangzhou 310022, China; 5 Jiangsu Key Lab of Cancer Biomarkers, Prevention and Treatment, Collaborative Innovation Center for Cancer Medicine and International Joint Research Center on Environment and Human Health, Nanjing Medical University, Nanjing 211166, China; 6 Department of Epidemiology, Fuwai Hospital, National Center for Cardiovascular Diseases, Chinese Academy of Medical Sciences and Peking Union Medical College, Beijing 100037, China; 7 Key Laboratory of Cardiovascular Epidemiology, Chinese Academy of Medical Sciences, Beijing 100037, China; 8 Research Units of Cohort Study on Cardiovascular Diseases and Cancers, Chinese Academy of Medical Sciences, Beijing 100730, China; 9 Department of General Surgery, The First Affiliated Hospital of Nanjing Medical University, Nanjing 210029, China; 10 Office of Cancer Screening, National Cancer Center/National Clinical Research Center for Cancer/Cancer Hospital, Chinese Academy of Medical Sciences and Peking Union Medical College, Beijing 100021, China

**Keywords:** obesity, metabolomics, colorectal cancer, cohort

## Abstract

Obesity is a leading contributor to colorectal cancer (CRC) risk, but the metabolic mechanisms linking obesity to CRC are not fully understood. We leveraged untargeted metabolomics data from two 1:1 matched, nested case–control studies for CRC, including 223 pairs from the US Prostate, Lung, Colorectal, and Ovarian (PLCO) Cancer Screening Trial and 190 pairs from a prospective Chinese cohort. We explored serum metabolites related to body mass index (BMI), constructed a metabolomic signature of obesity, and examined the association between the signature and CRC risk. In total, 72 of 278 named metabolites were correlated with BMI after multiple testing corrections (*p* FDR < 0.05). The metabolomic signature was calculated by including 39 metabolites that were independently associated with BMI. There was a linear positive association between the signature and CRC risk in both cohorts (*p* for linear < 0.05). Per 1-SD increment of the signature was associated with 38% (95% CI: 9–75%) and 28% (95% CI: 2–62%) higher risks of CRC in the US and Chinese cohorts, respectively. In conclusion, we identified a metabolomic signature for obesity and demonstrated the association between the signature and CRC risk. The findings offer new insights into the underlying mechanisms of CRC, which is critical for improved CRC prevention.

## 1. Introduction

As the third most common and second most deadly malignancy globally, colorectal cancer (CRC) caused over 1.9 million new cases and 0.9 million deaths in 2020 [1]. The burden is projected to increase to 3.2 million new cases and 1.6 million deaths by 2040 [2]. Obesity, a major public health concern worldwide, has been recognized as a leading risk factor for CRC [3]. Evidence from experimental and molecular epidemiologic studies indicates that obesity can cause chronic inflammation, dysregulation of sex hormones, and alterations in insulin signaling, thereby promoting the development of CRC [4]. Notably, obesity is a systemic disease featured with substantial metabolic and endocrine abnormalities [5]. A better understanding of metabolic disturbances underlying the association between obesity and CRC is crucial to develop effective strategies to mitigate future CRC risk.

Metabolomics has emerged as a powerful tool to identify novel biomarkers for metabolic characteristics and reveal mechanisms underlying complex diseases [6]. Previous metabolomic studies have reported multiple metabolites in relation to body mass index (BMI), including lipids, amino acids, peptides, and nucleotides [7,8,9,10,11,12,13]. Some of the studies further examined the association between the identified metabolites and the risks of diabetes [8], breast cancer [11], and prostate cancer [12]. In a nested case–control study within the European Prospective Investigation into Cancer and Nutrition (EPIC) cohort, a metabolomic signature comprising 31 lipids and 11 amino acids was established for BMI, but this signature was not statically significantly associated with CRC risk [14]. As acknowledged by the authors, the study was limited by the use of targeted metabolomics, which measured a set of metabolites of interest defined a priori [14]. Therefore, additional efforts are needed to uncover the metabolic effects of obesity on CRC risk.

Leveraging untargeted metabolomics data from a nested case–control study within the US Prostate, Lung, Colorectal, and Ovarian (PLCO) Cancer Screening Trial, we aimed to identify BMI-related metabolites and develop a metabolomic signature for BMI. We further evaluated the association of the metabolomic signature with CRC risk in the PLCO and validated this association through an independent nested case–control study based on a Chinese cohort. We also assessed the mediating effect of the signature on the association between BMI and CRC.

## 2. Materials and Methods

### 2.1. Study Design and Participants

The PLCO study is a randomized controlled trial to determine the effect of specific screening exams on reducing mortality from prostate, lung, colorectal, and ovarian cancers, which has been described previously [15]. Briefly, approximately 155,000 participants aged 55 to 74 years, who had no history of prostate, lung, colorectal, or ovarian cancer, were enrolled from ten US medical centers between 1993 and 2001 and were randomly assigned to the screened or the non-screened arm. All participants were asked to complete a baseline questionnaire collecting information on anthropometrics, demographics, lifestyle, and health status. Blood samples drawn at baseline were centrifuged into serum, plasma, red blood cells, and buffy coat fractions stored at −70  °C. The study was approved by the Institutional Review Boards at the National Cancer Institute and ten recruitment centers, and all participants provided informed consent.

The current analysis was based on a nested, 1:1 matched case–control study within the screening arm of the PLCO cohort. Participants were eligible if they had no self-reported history of cancer (except basal-cell skin cancer), Crohn’s disease, ulcerative colitis, familial polyposis, Gardner’s syndrome, or colorectal polyps at baseline and had been followed up for at least 6 months. Controls, who were free from any cancer at the time the matched case was diagnosed, were incidence–density sampled and matched to cases by age at randomization (5-year intervals), sex, race, year of randomization, and season of blood draw. Finally, 223 pairs of cases and controls that had available metabolomics data were included.

To validate results from the PLCO, we designed a 1:1 matched, nested case–control study for CRC based on a prospective cohort in Jiangsu Province, China. Details of the study design have been described elsewhere [16]. Briefly, a total of 44,962 adults completed an interviewer-administered electronic questionnaire and underwent physical examinations at baseline. Blood samples were collected after an overnight fast of at least 8 h and were immediately centrifuged into plasma, red blood cells, and white blood cells stored at −80 °C. Until December 2020, the overall follow-up rate was approximately 90%, and 190 incident CRC cases were recorded. The cancer diagnoses were confirmed by reviewing the cancer registration database and/or by visits to local communities. We selected controls randomly from cancer-free participants and matched them 1:1 to the incident CRC cases by age (±2 years), sex, and region. All participants provided written informed consent, and the study was approved by the Nanjing Medical University.

### 2.2. Metabolomic Profiling

In the PLCO, untargeted serum metabolomics data were generated by using the Metabolon Inc. platform consisting of ultra-high performance liquid chromatography-tandem mass spectrometry (UHPLC-MS/MS) and gas chromatography-mass spectrometry (GC-MS). The details of the procedures have been described elsewhere [17]. In brief, protein precipitation with methanol was performed to extract a broad coverage of metabolites in the serum. The extracts for UHPLC-MS/MS were analyzed on a Waters ACQUITY UPLC (Waters, Milford, MA, USA), and the extracts for GC-MS were analyzed on a Thermo-Finnigan Trace DSQ fast-scanning single-quadrupole MS (Thermo Finnegan, San Jose, CA, USA). Each batch contained up to 30 samples, including blinded quality-control samples of pooled serum at a level of 10%. Matched cases and controls were consecutively arranged in a counterbalanced order within each batch. In addition, a standard was spiked every six samples for quality control. The metabolites were identified by comparison to a chemical reference library generated from 2500 standards. A total of 447 named metabolites were identified, out of which 278 metabolites were measured in >80% of the participants and included in the analysis. These metabolites included amino acids, lipids, peptides, carbohydrates, cofactors and vitamins, xenobiotics, nucleotides, and energy.

In the Jiangsu cohort, untargeted metabolites in the plasma were measured using UHPLC-MS/MS at Metabolon, as described in detail elsewhere [16,18]. Briefly, based on ACQUITY UPLC (Waters, Milford, MA, USA) and Q Exactive HF hybrid Quadrupole-Orbitrap (Thermo Fisher Scientific, San Jose, CA, USA), four independent UHPLC-MS/MS methods were applied: two separate reverse-phase (RP)/UHPLC-MS/MS methods with positive-ion mode electrospray ionization (ESI), RP/UHPLC-MS/MS with negative-ion mode ESI, and hydrophilic interaction liquid chromatography (HILIC)/UHPLC-MS/MS with negative-ion mode ESI. The methods for quality control and metabolite identification were similar to those used in the PLCO.

### 2.3. Exposure and Covariate Measurement

Information on age, sex, smoking, alcohol drinking, history of diabetes, and family history of CRC in first-degree relatives was derived from structured baseline questionnaires. BMI was calculated as weight in kilograms divided by height in meters squared. In the US, BMI ≥ 30 kg/m^2^ was defined as obesity [19], and in China, the cut-off point was 28 kg/m^2^ [20]. Pack-years of smoking were calculated by multiplying the number of packs smoked per day by the number of years smoked. Alcohol consumed in grams per day was calculated by multiplying alcoholic beverage consumed (mL) with alcohol concentration (%) and alcohol density (0.8) by the frequency and usual serving size of alcohol consumption [21].

### 2.4. Statistical Analysis

In order to account for the potential batch effect and improve normality, metabolite concentrations were batch-normalized (divided by the batch median for each metabolite) and then transformed. Metabolite values below the limit of detection were assigned with the minimum of all observed values. Baseline characteristics between CRC cases and controls were compared by Chi-squared tests for categorical variables and Wilcoxon rank tests for continuous variables. In the PLCO, Spearman’s partial correlation was performed to estimate the correlations between baseline BMI and 278 metabolites, with adjustment for potential confounders, including age, sex, smoking status, and pack-years of smoking. In a secondary analysis, we analyzed the correlation of weight change from 20 years old to study baseline with the metabolites. The false discovery rate (FDR) was used for multiple testing corrections, with *p* FDR < 0.05 considered statistically significant [22]. We then performed the Least Absolute Shrinkage and Selection Operator (LASSO) analysis to select metabolites that were most informative of BMI [23]. The metabolomic signature was calculated as the weighted sum of the selected metabolites with weights equal to coefficients from the LASSO regression [24]. The percent variation in BMI explained by the metabolomic signature was assessed by R-squared from linear regression. We also applied random forest for feature selection and obtained variable importance to examine the robustness of the results from LASSO [25]. Because not all BMI-related metabolites included in the metabolomic signature (n = 39) were measured in the Jiangsu cohort (n = 27), we then fitted a re-weighted metabolomic signature using ridge regression [26,27]. Spearman’s correlation was used to assess the strength of the relationship between the re-weighted signature and the original signature.

We used multivariable restricted cubic splines with four knots (5th, 35th, 65th, and 95th percentiles) to explore the precise shape of the dose–response curve between the metabolomic signature and CRC risk. The likelihood ratio tests were performed to assess nonlinearity and linearity. *P* for nonlinear < 0.05 was defined as nonlinearity, while *p* for nonlinear > 0.05 and *p* for linear < 0.05 was defined as linearity. To evaluate the associations of the metabolomic signature and BMI-related metabolites with CRC risk, multivariable conditional logistic regression models were used to compute odds ratios (ORs) and 95% confidence intervals (CIs) by quartiles and per 1-standard deviation (SD) increment of the metabolomic signature and metabolites. Model 1 was adjusted for age (continuous), and Model 2 was further adjusted for smoking status (never, former, current), pack-years of smoking (continuous), alcohol intake (g/day), history of diabetes (yes, no), and in the PLCO, study center and family history of CRC (yes, no). The stratified analysis was conducted according to median age, sex, smoking status, and median time to CRC diagnosis. Potential interaction effects were assessed by including a product term between metabolite concentrations and the categorical stratified variable in Model 2.

To assess the mediation effect of the metabolomic signature on the association between BMI and CRC, we decomposed the “total effect” of BMI into an “indirect effect” (i.e., through metabolites) and a “direct effect” (i.e., through other mechanisms) [28]. The total and direct effects were estimated by multivariable logistic regression and presented as ORs and 95% CIs, without and with the metabolomic signature as a covariate. All statistical tests were two-sided and performed using SAS 9.4 (SAS Institute, Carry, NC, USA) and R 3.6.3 (R Foundation for Statistical Computing, Vienna, Austria).

## 3. Results

### 3.1. Population Characteristics

The median follow-up periods from blood collection to CRC diagnosis were 8.0 and 9.0 years in the PLCO and Jiangsu cohorts, respectively. In the PLCO, participants were, on average, 64.4 (SD = 5.1) years old at baseline, and 56.9% were male. The proportion of obesity was higher in CRC cases than in controls (29.7% vs. 20.0%, *p* = 0.02). In the Jiangsu cohort, the mean age of participants was 59.7 (SD = 10.6) years, and 55.8% were male. The proportions of obesity were 14.2% in cases and 6.3% in controls (*p* = 0.02) (Table 1).

### 3.2. Metabolites Correlated with BMI

**Figure 1:**
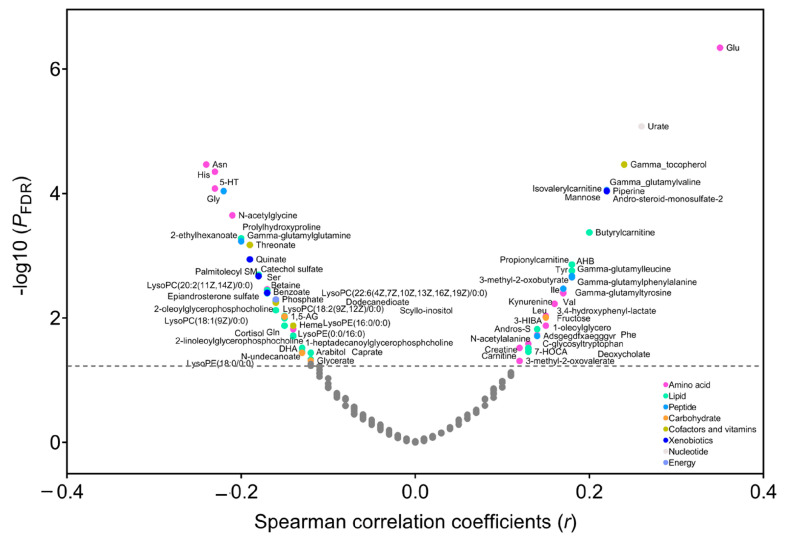
Correlation between metabolites and body mass index (BMI) in the PLCO. Spearman’s partial correlation was used to adjust for age, sex, smoking status, and pack-years of smoking. The metabolites above the horizontal line had a statistically significant correlation with BMI (*p* FDR < 0.05). Abbreviations: PLCO, Prostate, Lung, Colorectal, and Ovarian Cancer Screening Trial; FDR, false discovery rate.

In the PLCO, Spearman’s partial correlation showed that 33 metabolites were positively (*r* ranging from 0.35 to 0.12, *p* FDR < 0.05) and 39 metabolites were inversely correlated with BMI (*r* ranging from −0.24 to −0.12, *p* FDR < 0.05) (Figure 1 and Table S1). These metabolites included lipids (n = 27), amino acids (n = 23), peptides (n = 7), carbohydrates (n = 5), cofactors and vitamins (n = 4), xenobiotics (n = 4), nucleotide (n = 1), and energy (n = 1). Using LASSO, we identified that 39 of the 72 BMI-related metabolites were independent predictors of BMI (i.e., 14 lipids, 12 amino acids, 3 peptides, 2 carbohydrates, 3 cofactors and vitamins, 3 xenobiotics, 1 nucleotide, and 1 energy) (Figure S1), most of which also had a large variable importance score in the random forest model (Figure S2). Then we created a metabolomic signature based on the coefficients generated in LASSO (Table S2) and the concentrations of selected metabolites. The signature was highly correlated with BMI (*r* = 0.73, *p* < 0.0001) and explained 53% of the variation in BMI. In the secondary analysis, we found that 59 metabolites were correlated with weight change over a period from 20 years old to study baseline (*p* FDR < 0.05), 53 of which overlapped with BMI-related metabolites (n = 72) (Table S3). In addition, 56 of 72 BMI-related metabolites were available in the Jiangsu cohort, and we found that a total of 20 metabolites were validated to correlate with BMI in the Jiangsu cohort (nominal *p* < 0.05) (Figure S3).

### 3.3. Metabolomic Signature and CRC Risk

**Figure 2:**
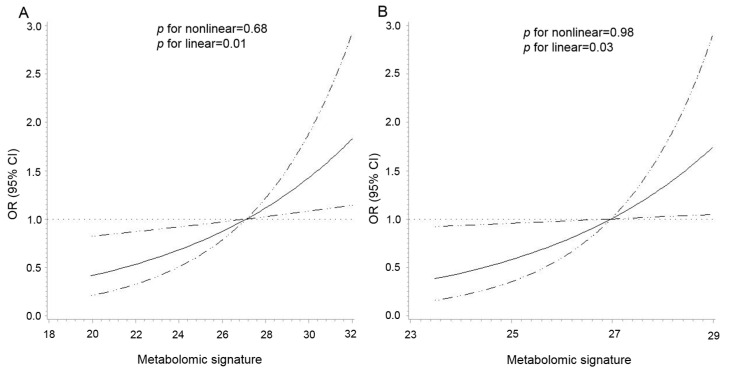
Dose–response association between the metabolomic signature of body mass index and colorectal cancer risk in the PLCO (**A**) and Jiangsu (**B**) cohorts. The association was examined by the multivariable conditional logistic model with restricted cubic splines. Solid lines represent estimates of odds ratio, and dashed lines represent 95% confidence intervals (*p* for linear = 0.01 in the PLCO; *p* for linear = 0.03 in the Jiangsu cohort). The metabolomic signature above 95% was not plotted due to wide confidence intervals at the extremes. Abbreviations: PLCO, Prostate, Lung, Colorectal, and Ovarian Cancer Screening Trial.

As shown in Figure 2, there was a positive linear association between the metabolomic signature and CRC risk in both cohorts (*p* for nonlinear = 0.68 and *p* for linear = 0.01 in the PLCO, *p* for nonlinear = 0.98 and *p* for linear = 0.03 in the Jiangsu cohort). The multivariable analysis showed that per 1-SD increment of the signature was associated with a 38% (95% CI: 9%–75%) higher risk of CRC (Table 2). The re-weighted metabolomic signature, including 27 available BMI-related metabolites in the Jiangsu cohort, showed excellent correspondence to the signature created in the PLCO (*r* = 0.92, *p* <0.0001). Per 1-SD increment in the re-weighted metabolomic signature was also associated with an increased risk of CRC in the Jiangsu cohort (OR = 1.28, 95% CI: 1.02–1.62) (Table 2). In the stratified analyses by age, sex, smoking status, and median time to diagnosis, the association between the metabolomic signature and CRC risk was similar across strata in both cohorts without any statistically significant modification effect (*p* for interaction > 0.05) (Figure S4).

For the association between each BMI-related metabolite and CRC risk, we found that glutamine (OR per 1-SD increment =0.72, 95% CI: 0.57–0.92), histidine (OR = 0.73, 95% CI: 0.57–0.92), and gamma-glutamyl glutamine (OR = 0.72, 95% CI: 0.56–0.93) were inversely, and andro steroid mono sulfate 2 was positively associated with CRC risk in the PLCO. Among the four metabolites, three were available in the Jiangsu cohort, and the negative association between glutamine and CRC risk was replicated (OR = 0.50, 95% CI: 0.28–0.90). Although the validation results were null for histidine and andro steroid mono sulfate 2, their association directions with CRC risk were consistent in both cohorts (Table S4).

### 3.4. Mediation Effect

**Figure 3:**
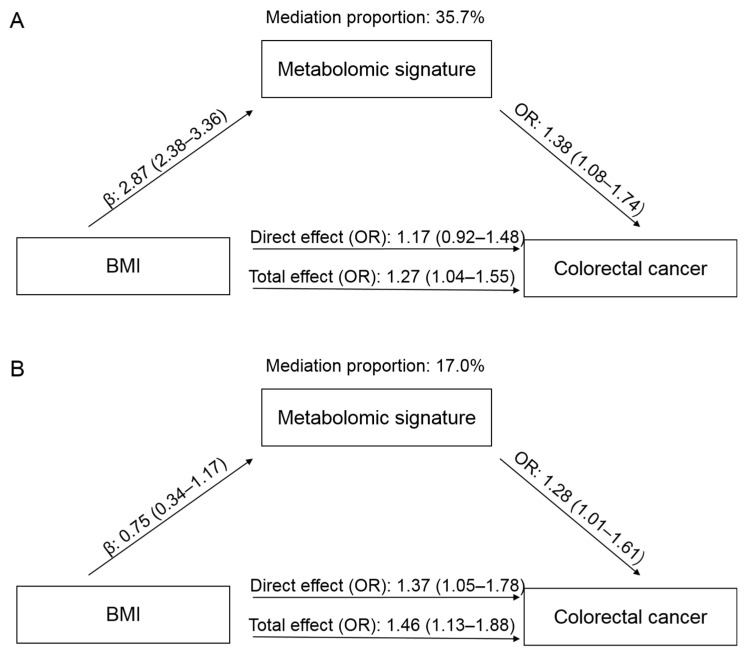
Mediation effects of the metabolomic signature on the association between body mass index (BMI) and colorectal cancer (CRC) in the PLCO (**A**) and Jiangsu (**B**) cohorts. The total effect represents the association between BMI and CRC without adjustment for metabolomic signature, while the direct effect shows the association after adjustment for metabolomic signature. Abbreviations: PLCO, Prostate, Lung, Colorectal, and Ovarian Cancer Screening Trial; OR, odds ratio.

In the PLCO, a higher BMI was associated with an increased risk of CRC, with an OR of 1.27 (95% CI: 1.04–1.55), comparing obesity to non-obesity (Figure 3). When adding the metabolomic signature into the model, the effect of BMI was attenuated to 1.17 (95% CI: 0.92–1.48). The mediation proportion of the signature was 35.7%. In the Jiangsu cohort, the OR for CRC comparing obesity to non-obesity was 1.46 (95% CI: 1.13–1.88), which was attenuated to 1.37 (95% CI: 1.05–1.78) when the metabolomic signature was added. The mediation proportion was 17.0%.

## 4. Discussion

Leveraging data from two nested case–control studies, we identified 72 BMI-related metabolites and created a metabolomic signature for BMI. The signature incorporated 39 metabolites belonging to amino acids, lipids, peptides, carbohydrates, cofactors and vitamins, xenobiotics, nucleotides, and energy. We found that the signature had a linear positive association with CRC risk and partially mediated the association between BMI and CRC in both US and Chinese populations. Therefore, our study provides a panel of blood metabolites reflective of widespread metabolic disturbances caused by obesity and sheds light on the underlying mechanisms of colorectal carcinogenesis. The created signature also holds the potential to improve the identification of individuals at high risk of CRC for early intervention.

To the best of our knowledge, 54 of 72 BMI-related metabolites identified in the current study have been reported in previous studies [11,13,29,30,31,32]. For example, consistent with our findings about amino acids, a metabolomic study based on the TwinsUK and Health Nucleus cohorts also showed positive correlations of BMI with serum levels of glutamate, N-acetylalanine, creatine, aromatic amino acids (C-glycosyltryptophan, tyrosine, and phenylalanine), and branched-chain amino acid (leucine, isoleucine, and valine), as well as inverse correlations with glutamine, asparagine, serotonin, histidine, and several metabolites involved in glycine, serine and threonine metabolism [13]. Moreover, a two-sample mendelian randomization study supported the causal effects of BMI on aromatic amino acids, branched-chain amino acids, and glutamine [33]. The increase of aromatic amino acids and branched-chain amino acids in obese individuals is thought to be related to liver dysfunction in catalyzing the metabolites and abnormal expression of amino acid catabolic genes in adipose tissue [29]. Additionally, the reduction of glycine levels with obesity is likely attributed to decreased gut absorption, weakened biosynthesis, and increased catabolism or urine excretion [34].

In addition to amino acids, lipid derivatives represent another major group of metabolites associated with BMI. In line with our results, several observational studies have reported that BMI was positively correlated with serum levels of androsteroid mono sulfate 2, carnitine, bile acid derivates, 2-hydroxybutyrate, and 1-oleoylglycero, and was inversely correlated with androgenic steroids (epiandrosterone sulfate and androsterone sulfate), medium chain fatty acids, glycerophospholipid metabolites, palmitoyl sphingomyelin, cortisol, and docosahexaenoic [11,13,30]. Adipose tissue is recognized as an important site for the synthesis, metabolism, and storage of steroid hormones [35]. Functional impairments of adipose tissue in obese individuals could lead to the imbalance of steroid biosynthesis and other lipid perturbations [36]. Most of the other BMI-related metabolites in groups of peptides, carbohydrates, cofactors and vitamins, xenobiotics, nucleotides, and energy also have been reported in previous studies [11,13,31,32]. Besides, we identified 18 novel BMI-related metabolites, including 2 amino acids, 11 lipids, 2 peptides, 2 xenobiotics, and heme, thus providing more comprehensive metabolic disturbances present in obesity.

To further understand the role of obesity-related metabolic alterations in CRC development, we established a metabolomic signature of BMI and demonstrated its positive association with CRC risk. In a previous nested case–control study including 423 pairs of CRC cases and controls from the EPIC cohort, there was a marginally significant positive association between a BMI-related metabolomic signature and CRC risk (OR per 1-SD = 1.16, 95% CI: 0.99–1.35) before the adjustment for anthropometric measures including BMI [14]. Compared with the new signature in our study covering multiple metabolic pathways, the EPIC signature was only enriched in lipids and amino acids, which might not sufficiently reflect the effect of obesity on CRC development. A previous study reported that the inclusion of age, sex, high-density lipoprotein, low-density lipoprotein, total cholesterol, and triglycerides could explain 31% of the variance in BMI [13], while the metabolomic signature in our study could explain 53%. Therefore, compared with conventional clinical indicators, a metabolomic signature might provide additional information and be used to identify individuals at high risk of CRC.

Among BMI-related amino acids, we observed that glutamine and histidine were inversely associated with CRC risk in the PLCO. The results are in line with a nested case–control study of multiple cancer types, including CRC within the EPIC cohort, supporting that a reduction in the glutamine and histidine levels may precede CRC development [37]. Two hospital-based case–control studies also reported lower serum levels of glutamine and histidine in CRC patients compared to healthy controls [38,39]. It has been proposed that glutamine is a trophic and cytoprotective factor of the intestinal mucosa, which may preserve mucosal integrity, maintain intestinal barrier function, and enhance intestinal immunity [40,41]. For histidine, experimental studies have shown its anti-inflammatory effect on intestinal epithelial cells by suppressing nuclear factor-kappa B activation and proinflammatory cytokine production [42,43].

Moreover, our analysis in the PLCO identified a positive association of andro steroid mono sulfate 2, an androgen metabolite in serum, with CRC risk. In support of the result, a nested case–control study of Japanese postmenopausal women reported a positive association between total plasma testosterone and CRC risk [44]. Testosterone has the potential to stimulate the growth of colon cancer cells in vitro, and the effect could be inhibited by anti-androgens [45]. The exact function of andro steroid mono sulfate 2 remains largely unknown, and future studies are required to better understand its role in CRC development. To the best of our knowledge, this study is the first observed inverse association between gamma-glutamyl glutamine and CRC risk. Gamma-glutamyl glutamine belongs to the class of organic compounds known as dipeptides. Gamma-glutamyl peptides play a role in various physiological functions, including anti-inflammatory and antioxidant effects [46]. However, the role of gamma-glutamyl glutamine in colorectal carcinogenesis needs further investigation.

Our study has several strengths, including the prospective design, untargeted metabolomics approach covering a wide range of metabolites, and an independent validation for the association between the metabolic signature and CRC risk. However, we also acknowledge several limitations. First, BMI was calculated from self-reported height and weight in the PLCO, which might introduce information bias. Meanwhile, we lacked data on other indicators of excess body fat. Second, the correlation analysis for BMI and metabolites was cross-sectional, limiting causal inference. However, the majority of identified metabolites are consistent with those reported in previous epidemiologic studies and supported by biological evidence. Third, metabolomic profiling was conducted only once in each cohort, and an individual’s metabolite levels may vary over time. However, previous metabolomic studies of repeated assessments showed that the majority of metabolites in the blood were stable over at least four years [47,48].

In conclusion, based on two nested case–control studies, we identified a metabolomic signature of BMI involving multiple metabolic pathways and demonstrated its positive association with CRC risk. Our study provides novel insights into the mechanisms underlying the obesity–CRC association and informs future research to better identify individuals at high risk of CRC. Future studies are warranted to uncover metabolic targets and approaches for improved prevention of CRC.

## Figures and Tables

**Table 1:** Baseline characteristics of colorectal cancer cases and controls from the PLCO and Jiangsu cohorts.

Characteristic ^a^	PLCO			Jiangsu		
	Case (n = 223)	Control (n = 223)	*p* ^b^	Case (n = 190)	Control (n = 190)	*p* ^b^
Age, years	64.3 (5.2)	64.4 (5.1)	0.95	59.7 (10.6)	59.7 (10.6)	0.98
Female, %	43.1	43.1	–	44.2	44.2	–
Smoking status, %			0.61			0.07
Never	41.3	43.5		60.0	65.3	
Former	47.9	48.4		6.8	2.1	
Current	10.8	8.1		33.2	32.6	
Pack-years of smoking	18.8 (25.2)	18.0 (27.1)	0.44	12.3 (20.3)	10.8 (19.7)	0.31
Height, cm	171.7 (10.2)	171.5 (9.4)	0.85	160.9 (8.7)	160.5 (8.4)	0.74
Body weight, kg	81.8 (17.3)	78.4 (16.7)	0.04	60.8 (10.5)	60.8 (10.3)	0.83
BMI, kg/m^2^	27.9 (4.7)	26.7 (4.6)	0.01	23.8 (3.7)	23.6 (3.1)	0.69
Adiposity, % ^c^	29.7	20.0	0.02	14.2	6.3	0.02
Alcohol, g/day	12.2 (22.9)	12.8 (22.3)	0.38	17.4 (43.6)	13.0 (30.8)	0.69
Prevalence of diabetes, %	8.5	6.8	0.48	4.2	5.8	0.36
Family history of colorectal cancer, %	13.1	10.0	0.58	–	–	–

^a^ Values are mean (standard deviation) for continuous variables and percentages for categorical variables. ^b^ Chi-squared tests for categorical variables and Wilcoxon rank tests for continuous variables. ^c^ Obesity was defined as BMI ≥ 30 kg/m^2^ in the PLCO and BMI ≥ 28 kg/m^2^ in the Jiangsu cohort. Abbreviations: PLCO, Prostate, Lung, Colorectal, and Ovarian Cancer Screening Trial; BMI, body mass index.

**Table 2:** Association between the metabolomic signature of obesity and colorectal cancer risk in the PLCO and Jiangsu cohorts.

Cohort		Quartiles of Metabolomic Signature, OR (95% CI)				*p* for Trend	OR per 1-SD Increase
		Q1	Q2	Q3	Q4		
PLCO	No. of cases	47	53	59	64		
	Age-adjusted model	1 (referent)	1.31 (0.75–2.28)	1.60 (0.93–2.77)	2.01 (1.13–3.57)	0.01	1.34 (1.09–1.65)
	Multivariable model ^**a**^	1 (referent)	1.46 (0.81–2.65)	1.82 (1.00–3.30)	2.21 (1.15–4.25)	0.01	1.38 (1.09–1.75)
Jiangsu	No. of cases	41	45	50	54		
	Age-adjusted model	1 (referent)	1.27 (0.72–2.24)	1.52 (0.86–2.69)	1.87 (1.02–3.45)	0.01	1.34 (1.07–1.67)
	Multivariable model ^**a**^	1 (referent)	1.17 (0.65–2.12)	1.35 (0.74–2.46)	1.70 (0.90–3.19)	0.04	1.28 (1.02–1.62)

**^a^** Conditional logistic regression model adjusted for age, smoking status, pack-years of smoking, alcohol intake, diabetes history, and in the PLCO, study center and family history of colorectal cancer. Abbreviations: PLCO, Prostate, Lung, Colorectal, and Ovarian Cancer Screening Trial; OR, odds ratio; CI, confidence interval; SD, standard deviation.

## Data Availability

The PLCO data can be applied online at https://biometry.nci.nih.gov/cdas/plco/ (accessed on 3 February 2023). Other data are available from the corresponding author upon request due to privacy.

## Supplementary Materials

The following supporting information can be downloaded at: https://www.mdpi.com/article/10.3390/metabo13020234/s1, Figure S1: Selection of the optimal metabolites used to construct the metabolomic signature of body mass index by the LASSO (A and B) and Ridge regression models (C and D). The mean-squared error was plotted against log (λ), where λ was selected by ten-fold cross-validation; Figure S2: Importance of BMI-related metabolites based on the random forest model; Figure S3: Validation of BMI-related metabolites identified from the PLCO cohort among participants from the Jiangsu cohort; Figure S4: Stratified analysis of the association between the metabolomic signature and colorectal cancer risk by median age (PLCO: 64 years, Jiangsu: 60 years), sex (male, female), and median time to diagnosis (PLCO: 8.0 years, Jiangsu: 9.0 years); Table S1: The 72 metabolites correlated with body mass index among 446 participants from the PLCO cohort; Table S2: The coefficients from the LASSO and Ridge regression models; Table S3: The 59 metabolites correlated with weight change from 20 years old to baseline among 446 participants from the PLCO cohort; Table S4: Associations between BMI-related metabolites and colorectal cancer in the PLCO and Jiangsu cohorts.

## References

[1] Sung H., Ferlay J., Siegel R.L., Laversanne M., Soerjomataram I., Jemal A., Bray F. (2021). Global Cancer Statistics 2020: GLOBOCAN Estimates of Incidence and Mortality Worldwide for 36 Cancers in 185 Countries. CA Cancer J. Clin..

[2] Morgan E., Arnold M., Gini A., Lorenzoni V., Cabasag C.J., Laversanne M., Vignat J., Ferlay J., Murphy N., Bray F. (2023). Global burden of colorectal cancer in 2020 and 2040: Incidence and mortality estimates from GLOBOCAN. Gut.

[3] Lauby-Secretan B., Scoccianti C., Loomis D., Grosse Y., Bianchini F., Straif K. (2016). Body Fatness and Cancer—Viewpoint of the IARC Working Group. N. Engl. J. Med..

[4] Murphy N., Jenab M., Gunter M.J. (2018). Adiposity and gastrointestinal cancers: Epidemiology, mechanisms and future directions. Nat. Rev. Gastroenterol. Hepatol..

[5] Smith G.I., Mittendorfer B., Klein S. (2019). Metabolically healthy obesity: Facts and fantasies. J. Clin. Investig..

[6] Peng B., Li H., Peng X.X. (2015). Functional metabolomics: From biomarker discovery to metabolome reprogramming. Protein Cell.

[7] Ho J.E., Larson M.G., Ghorbani A., Cheng S., Chen M.-H., Keyes M., Rhee E.P., Clish C.B., Vasan R.S., Gerszten R.E. (2016). Metabolomic Profiles of Body Mass Index in the Framingham Heart Study Reveal Distinct Cardiometabolic Phenotypes. PLoS ONE.

[8] Cheng S., Rhee E.P., Larson M.G., Lewis G.D., McCabe E.L., Shen D., Palma M.J., Roberts L.D., Dejam A., Souza A.L. (2012). Metabolite profiling identifies pathways associated with metabolic risk in humans. Circulation.

[9] Moore S.C., Matthews C.E., Sampson J.N., Stolzenberg-Solomon R.Z., Zheng W., Cai Q., Tan Y.T., Chow W.-H., Ji B.-T., Liu D.K. (2014). Human metabolic correlates of body mass index. Metabolomics.

[10] Ottosson F., Brunkwall L., Ericson U., Nilsson P.M., Almgren P., Fernandez C., Melander O., Orho-Melander M. (2018). Connection Between BMI-Related Plasma Metabolite Profile and Gut Microbiota. J. Clin. Endocrinol. Metab..

[11] Moore S.C., Playdon M.C., Sampson J.N., Hoover R.N., Trabert B., E Matthews C., Ziegler R.G. (2018). A Metabolomics Analysis of Body Mass Index and Postmenopausal Breast Cancer Risk. J. Natl. Cancer Inst..

[12] Dickerman B.A., Ebot E.M., Healy B.C., Wilson K.M., Eliassen A.H., Ascherio A., Pernar C.H., Zeleznik O.A., Heiden M.G.V., Clish C.B. (2020). A Metabolomics Analysis of Adiposity and Advanced Prostate Cancer Risk in the Health Professionals Follow-Up Study. Metabolites.

[13] Cirulli E.T., Guo L., Swisher C.L., Shah N., Huang L., Napier L.A., Kirkness E.F., Spector T.D., Caskey C.T., Thorens B. (2019). Profound Perturbation of the Metabolome in Obesity Is Associated with Health Risk. Cell Metab..

[14] Kliemann N., Viallon V., Murphy N., Beeken R.J., Rothwell J.A., Rinaldi S., Assi N., van Roekel E.H., Schmidt J.A., Borch K.B. (2021). Metabolic signatures of greater body size and their associations with risk of colorectal and endometrial cancers in the European Prospective Investigation into Cancer and Nutrition. BMC Med..

[15] Prorok P.C., Andriole G.L., Bresalier R.S., Buys S.S., Chia D., Crawford E.D., Fogel R., Gelmann E.P., Gilbert F., Hasson M.A. (2000). Design of the prostate, lung, colorectal and ovarian (PLCO) cancer screening trial. Control. Clin. Trials.

[16] Hang D., Yang X., Lu J., Shen C., Dai J., Lu X., Jin G., Hu Z., Gu D., Ma H. (2022). Untargeted plasma metabolomics for risk prediction of hepatocellular carcinoma: A prospective study in two Chinese cohorts. Int. J. Cancer.

[17] Evans A.M., DeHaven C.D., Barrett T., Mitchell M., Milgram E. (2009). Integrated, nontargeted ultrahigh performance liquid chromatography/electrospray ionization tandem mass spectrometry platform for the identification and relative quantification of the small-molecule complement of biological systems. Anal. Chem..

[18] Shen B., Yi X., Sun Y., Bi X., Du J., Zhang C., Quan S., Zhang F., Sun R., Qian L. (2020). Proteomic and Metabolomic Characterization of COVID-19 Patient Sera. Cell.

[19] Pi-Sunyer F.X. (2000). Obesity: Criteria and classification. Proc. Nutr. Soc..

[20] Zhou B.F., Cooperative Meta-Analysis Group of the Working Group on Obesity in China (2002). Predictive values of body mass index and waist circumference for risk factors of certain related diseases in Chinese adults—Study on optimal cut-off points of body mass index and waist circumference in Chinese adults. Biomed. Environ. Sci..

[21] Troy J.D., Hartge P., Weissfeld J.L., Oken M.M., Colditz G.A., Mechanic L.E., Morton L.M. (2010). Associations between anthropometry, cigarette smoking, alcohol consumption, and non-Hodgkin lymphoma in the Prostate, Lung, Colorectal, and Ovarian Cancer Screening Trial. Am. J. Epidemiol..

[22] Benjamini Y., Hochberg Y. (1995). Controlling the False Discovery Rate: A Practical and Powerful Approach to Multiple Testing. J. R. Stat. Soc. Ser. B.

[23] Tibshirani R. (1996). Regression Shrinkage and Selection Via the Lasso. J. R. Stat. Soc. Ser. B.

[24] Li J., Guasch-Ferré M., Chung W., Ruiz-Canela M., Toledo E., Corella D., Bhupathiraju S., Tobias D., Tabung F., Hu J. (2020). The Mediterranean diet, plasma metabolome, and cardiovascular disease risk. Eur. Heart J..

[25] Aleksandrova K., Reichmann R., Kaaks R., Jenab M., Bueno-De-Mesquita H.B., Dahm C.C., Eriksen A.K., Tjønneland A., Artaud F., Boutron-Ruault M.-C. (2021). Development and validation of a lifestyle-based model for colorectal cancer risk prediction: The LiFeCRC score. BMC Med..

[26] Murthy V.L., Reis J.P., Pico A.R., Kitchen R., Lima J.A., Lloyd-Jones D., Allen N.B., Carnethon M., Lewis G.D., Nayor M. (2020). Comprehensive Metabolic Phenotyping Refines Cardiovascular Risk in Young Adults. Circulation.

[27] Arthur E., Hoerl Robert W., Technometrics K.J. (2000). Ridge Regression: Biased Estimation for Nonorthogonal Problems. Technometrics.

[28] Vanderweele T.J., Vansteelandt S. (2010). Odds ratios for mediation analysis for a dichotomous outcome. Am. J. Epidemiol..

[29] Payab M., Tayanloo-Beik A., Falahzadeh K., Mousavi M., Salehi S., Djalalinia S., Ebrahimpur M., Rezaei N., Rezaei-Tavirani M., Larijani B. (2022). Metabolomics prospect of obesity and metabolic syndrome; a systematic review. J. Diabetes Metab. Disord..

[30] Butte N.F., Liu Y., Zakeri I.F., Mohney R.P., Mehta N.R., Voruganti V.S., Goring H.H.H., Cole S.A., Comuzzie A.G. (2015). Global metabolomic profiling targeting childhood obesity in the Hispanic population. Am. J. Clin. Nutr..

[31] Kim M.J., Yang H.J., Kim J.H., Ahn C.W., Lee J.H., Kim K.S., Kwon D.Y. (2013). Obesity-related metabolomic analysis of human subjects in black soybean peptide intervention study by ultraperformance liquid chromatography and quadrupole-time-of-flight mass spectrometry. J. Obes..

[32] Celik N., Andiran N. (2011). The relationship between serum phosphate levels with childhood obesity and insulin resistance. J. Pediatr. Endocrinol. Metab. JPEM.

[33] Bull C.J., Bell J.A., Murphy N., Sanderson E., Smith G.D., Timpson N.J., Banbury B.L., Albanes D., Berndt S.I., Bézieau S. (2020). Adiposity, metabolites, and colorectal cancer risk: Mendelian randomization study. BMC Med..

[34] Alves A., Bassot A., Bulteau A.L., Pirola L., Morio B. (2019). Glycine Metabolism and Its Alterations in Obesity and Metabolic Diseases. Nutrients.

[35] Li J., Daly E., Campioli E., Wabitsch M., Papadopoulos V. (2014). De novo synthesis of steroids and oxysterols in adipocytes. J. Biol. Chem..

[36] O’Flanagan C.H., Bowers L.W., Hursting S.D. (2015). A weighty problem: Metabolic perturbations and the obesity-cancer link. Horm. Mol. Biol. Clin. Investig..

[37] Breeur M., Ferrari P., Dossus L., Jenab M., Johansson M., Rinaldi S., Travis R.C., His M., Key T.J., Schmidt J.A. (2022). Pan-cancer analysis of pre-diagnostic blood metabolite concentrations in the European Prospective Investigation into Cancer and Nutrition. BMC Med..

[38] Tan B., Qiu Y., Zou X., Chen T., Xie G., Cheng Y., Dong T., Zhao L., Feng B., Hu X. (2013). Metabonomics identifies serum metabolite markers of colorectal cancer. J. Proteome Res..

[39] Zhu J., Djukovic D., Deng L., Gu H., Himmati F., Chiorean E.G., Raftery D. (2014). Colorectal cancer detection using targeted serum metabolic profiling. J. Proteome Res..

[40] Wang W.W., Qiao S.Y., Li D.F. (2009). Amino acids and gut function. Amino Acids.

[41] Wu G., Wu Z., Dai Z., Yang Y., Wang W., Liu C., Wang B., Wang J. (2013). Dietary requirements of “nutritionally non-essential amino acids” by animals and humans. Amino Acids.

[42] Son D.O., Satsu H., Shimizu M. (2005). Histidine inhibits oxidative stress- and TNF-alpha-induced interleukin-8 secretion in intestinal epithelial cells. FEBS Lett..

[43] Andou A., Hisamatsu T., Okamoto S., Chinen H., Kamada N., Kobayashi T., Hashimoto M., Okutsu T., Shimbo K., Takeda T. (2009). Dietary histidine ameliorates murine colitis by inhibition of proinflammatory cytokine production from macrophages. Gastroenterology.

[44] Mori N., Sawada N., Iwasaki M., Yamaji T., Goto A., Shimazu T., Inoue M., Murphy N., Gunter M.J., Tsugane S. (2019). Circulating sex hormone levels and colorectal cancer risk in Japanese postmenopausal women: The JPHC nested case-control study. Int. J. Cancer.

[45] Tutton P.J., Barkla D.H. (1982). The influence of androgens, anti-androgens, and castration on cell proliferation in the jejunal and colonic crypt epithelia, and in dimethylhydrazine-induced adenocarcinoma of rat colon. Virchows Arch. B Cell Pathol. Incl. Mol. Pathol..

[46] Guha S., Majumder K. (2022). Comprehensive Review of γ-Glutamyl Peptides (γ-GPs) and Their Effect on Inflammation Concerning Cardiovascular Health. J. Agric. Food Chem..

[47] Townsend M.K., Clish C.B., Kraft P., Wu C., Souza A.L., ADeik A., Tworoger S.S., Wolpin B.M. (2013). Reproducibility of metabolomic profiles among men and women in 2 large cohort studies. Clin. Chem..

[48] Chen L., Wang D., Garmaeva S., Kurilshikov A., Vila A.V., Gacesa R., Sinha T., Segal E., Weersma R.K., Wijmenga C. (2021). The long-term genetic stability and individual specificity of the human gut microbiome. Cell.

