# Supplementary material for: A Metabolomic Signature of Obesity and Risk of Colorectal Cancer: Two Nested Case–Control Studies
Source: Metabolites. 2023 Feb 5;13(2):234. doi: 10.3390/metabo13020234 (PMC9965372; doi:10.3390/metabo13020234)
Supplement: Supplementary file 1 [file metabolites-13-00234-s001.zip › metabolites-2140368-supplementary.pdf]

Supplementary Material

A metabolomic signature of obesity and risk of colorectal cancer: two nested case-control studies

Mingjia Yang, Chen Zhu, Lingbin Du, Jianv Huang, Jiayi Lu, Jing Yang, Ye Tong, Meng Zhu, Ci Song, Chong Shen, Juncheng Dai, Xiangfeng Lu, Zekuan Xu, Ni Li, Hongxia Ma, Zhibin Hu, Dongfeng Gu, Guangfu Jin, Dong Hang, Hongbing Shen

Contents

Supplementary figures

Supplementary tables

Supplementary figures

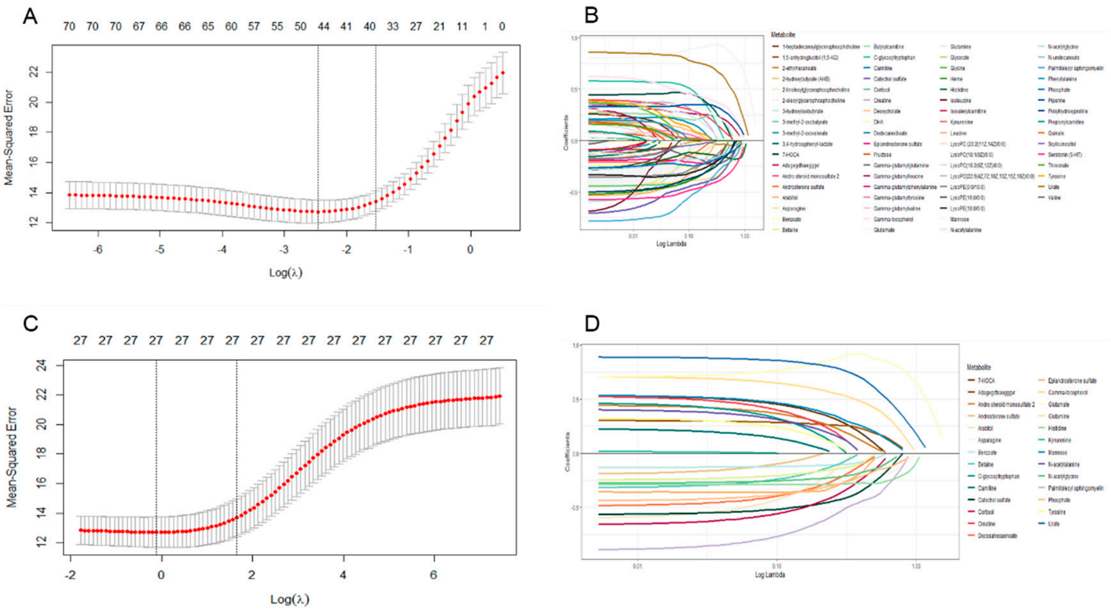

Figure S1. Selection of the optimal metabolites used to construct the metabolomic signature of body mass index by the LASSO (A and B) and Ridge regression models (C and D). The mean-squared error was plotted against log (λ), where λ was selected by ten-fold cross-validation. Abbreviations: LASSO, Least Absolute Shrinkage and Selection Operator.

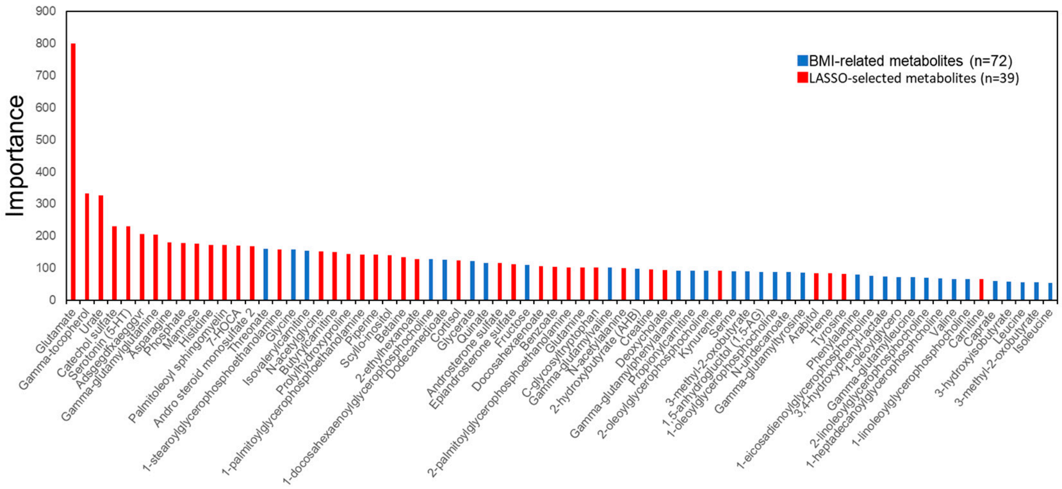

Figure S2. Importance of BMI-related metabolites based on the random forest model. Abbreviations: BMI, body mass index; LASSO, Least Absolute Shrinkage and Selection Operator.

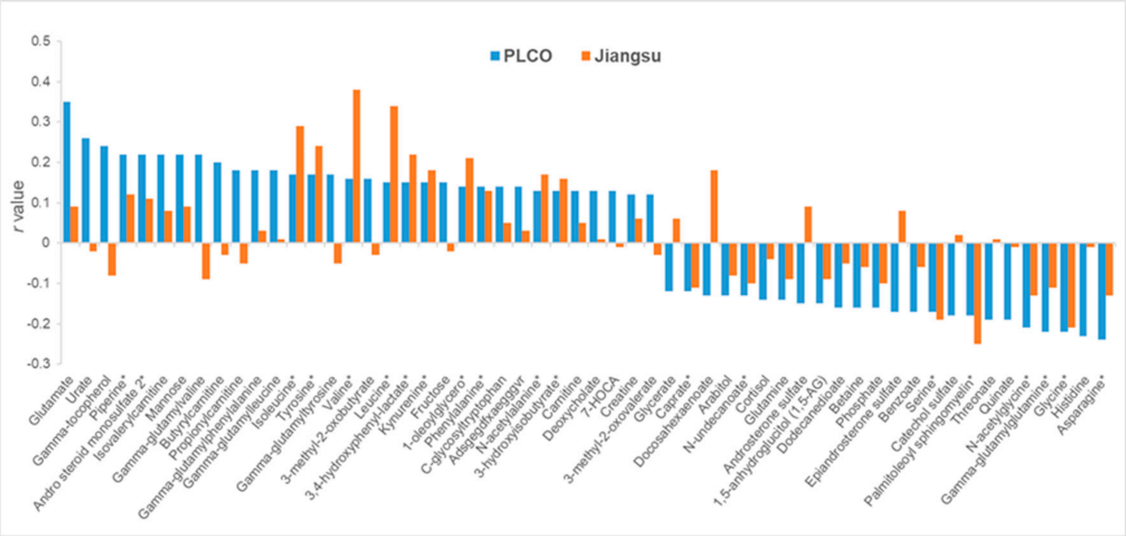

Figure S3. Validation of BMI-related metabolites identified from the PLCO cohort among participants from the Jiangsu cohort. A total of 20 metabolites (marked by asterisks) were validated to correlate with BMI in the Jiangsu cohort (nominal $p < 0.05$). Abbreviations: PLCO, Prostate, Lung, Colorectal, and Ovarian Cancer Screening Trial.

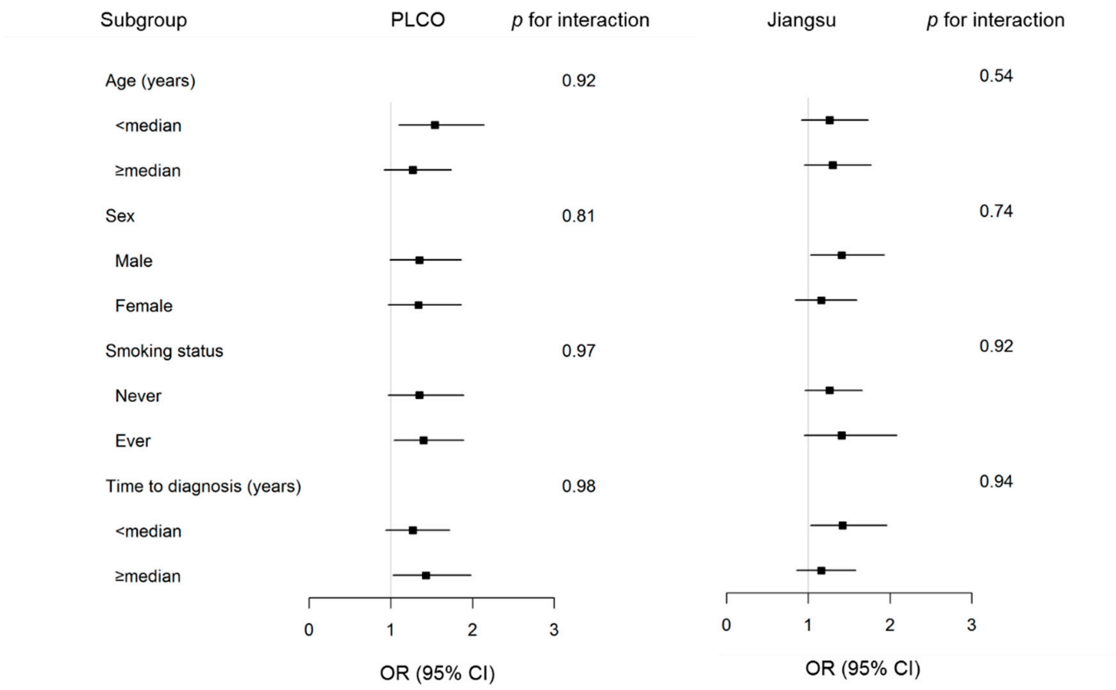

Figure S4. Stratified analysis of the association between the metabolomic signature and colorectal cancer risk by median age (PLCO: 64 years, Jiangsu: 60 years), sex (male, female), and median time to diagnosis (PLCO: 8.0 years, Jiangsu: 9.0 years). Abbreviations: PLCO, Prostate, Lung, Colorectal, and Ovarian Cancer Screening Trial

Supplementary tables

Table S1. The 72 metabolites correlated with body mass index among 446 participants from the PLCO cohort[a]

Metabolite	Sub pathway	HMDB	*r*	*p*	*p* FDR	Reported study (PMID)
Amino Acid						
Isovalerylcarnitine[b]	Leucine, isoleucine and valine metabolism	HMDB0000688	0.22	<0.001	<0.001	30318341
Isoleucine[b]	Leucine, isoleucine and valine metabolism	HMDB0000172	0.17	<0.001	<0.01	30318341 and 35673462
Valine	Leucine, isoleucine and valine metabolism	HMDB0000883	0.16	<0.001	<0.01	30318341 and 35673462
3-methyl-2-oxobutyrate[b]	Leucine, isoleucine and valine metabolism	HMDB0000019	0.16	<0.001	<0.01	30318341
Leucine	Leucine, isoleucine and valine metabolism	HMDB0000687	0.15	<0.01	0.01	30318341 and 35673462
3-hydroxyisobutyrate[b]	Leucine, isoleucine and valine metabolism	HMDB0000023	0.13	<0.01	0.03	NA
3-methyl-2-oxovalerate	Leucine, isoleucine and valine metabolism	HMDB0000491	0.12	0.01	0.05	30318341
Glycine[b]	Glycine, serine and threonine metabolism	HMDB0000123	-0.22	<0.001	<0.001	30318341 and 35673462
N-acetylglycine[b]	Glycine, serine and threonine metabolism	HMDB0000532	-0.21	<0.001	<0.001	30318341
Serine[b]	Glycine, serine and threonine metabolism	HMDB0000187	-0.17	<0.001	<0.01	30318341 and 35673462

Betaine[b]	Glycine, serine and threonine metabolism	HMDB0000043	-0.16	<0.001	<0.01	30318341 and 35673462
Serotonin (5-HT)[b]	Tryptophan metabolism	HMDB00259	-0.23	<0.001	<0.001	30318341 and 35673462
Kynurenine[b]	Tryptophan metabolism	HMDB0000684	0.15	<0.01	<0.01	30318341 and 35673462
C-glycosyltryptophan	Tryptophan metabolism	HMDB0240296	0.14	<0.01	0.02	30318341
Glutamate[b]	Glutamate metabolism	HMDB0000148	0.35	<0.001	<0.001	30318341 and 35673462
Glutamine[b]	Glutamate metabolism	HMDB0000641	-0.14	<0.01	0.02	30318341 and 35673462
Asparagine[b]	Alanine and aspartate metabolism	HMDB0000168	-0.24	<0.001	<0.001	30318341 and 35673462
N-acetylalanine	Alanine and aspartate metabolism	HMDB0000766	0.13	<0.01	0.03	30318341
Tyrosine[b]	Tyrosine metabolism	HMDB0000158	0.17	<0.001	<0.01	30318341 and 35673462
3,4-hydroxyphenyl-lactate	Tyrosine metabolism	HMDB0000755	0.15	<0.01	<0.01	NA
Histidine[b]	Histidine metabolism	HMDB0000177	-0.23	<0.001	<0.001	30318341 and 35673462
Phenylalanine	Phenylalanine metabolism	HMDB0000159	0.14	<0.01	0.02	30318341 and 35673462
Creatine[b]	Creatine metabolism	HMDB0000064	0.12	0.01	0.04	30318341 and 35673462
Lipid						
LysoPC(20:2(11Z,14Z)/0:0)[b]	Glycerophospholipid metabolism	HMDB10392	-0.17	<0.001	<0.01	NA
2-oleoylglycerophosphocholine[b]	Glycerophospholipid metabolism	NA	-0.16	<0.001	<0.01	26085512
LysoPC(22:6(4Z,7Z,10Z,13Z,16Z,19Z)/0:0)[b]	Glycerophospholipid metabolism	HMDB10404	-0.16	<0.001	<0.01	NA
LysoPC(18:2(9Z,12Z)/0:0)[b]	Glycerophospholipid metabolism	HMDB0010386	-0.16	<0.01	<0.01	NA
LysoPC(18:1(9Z)/0:0)[b]	Glycerophospholipid	HMDB02815	-0.15	<0.01	<0.01	NA

	metabolism					
LysoPE(16:0/0:0)[b]	Glycerophospholipid metabolism	HMDB11503	-0.15	<0.01	0.01	NA
2-linoleoylglycerophosphocholine[b]	Glycerophospholipid metabolism	NA	-0.14	<0.01	0.02	NA
LysoPE(0:0/16:0)	Glycerophospholipid metabolism	HMDB0011473	-0.14	<0.01	0.02	NA
1-heptadecanoylglycerophosphcholine[b]	Glycerophospholipid metabolism	NA	-0.13	<0.01	0.03	NA
LysoPE(18:0/0:0)	Glycerophospholipid metabolism	HMDB0011130	-0.12	0.01	0.05	NA
Andro steroid monosulfate 2[b]	Androgenic steroids	NA	0.22	<0.001	<0.001	26085512
Epiandrosterone sulfate[b]	Androgenic steroids	HMDB0062657	-0.17	<0.001	<0.01	26085512
Androsterone sulfate	Androgenic steroids	HMDB0002759	-0.15	<0.01	0.01	26085512
2-ethylhexanoate[b]	Medium chain fatty acid	HMDB0062546	-0.20	<0.001	<0.001	NA
N-undecanoate	Medium chain fatty acid	HMDB0000947	-0.13	<0.01	0.03	NA
Caprate	Medium chain fatty acid	HMDB0000511	-0.12	<0.01	0.04	30318341 and 26085512
Butyrylcarnitine[b]	Fatty acid metabolism (also BCAA metabolism)	HMDB0002013	0.20	<0.001	<0.001	30318341 and 26085512 and 29325144
Propionylcarnitine[b]	Fatty acid metabolism (also BCAA metabolism)	HMDB0000824	0.18	<0.001	<0.01	30318341 and 26085512
Deoxycholate	Bile acids, alcohols and derivatives	HMDB0246256	0.13	<0.01	0.03	30318341
7-HOCA[b]	Bile acids, alcohols and derivatives	HMDB0012458	0.13	<0.01	0.03	30318341 and 29325144
2-hydroxybutyrate (AHB)[b]	Glutathione metabolism	HMDB0000008	0.18	<0.001	<0.01	30318341

Palmitoleoyl sphingomyelin[b]	Sphingomyelins	HMDB0010169	-0.18	<0.001	<0.01	29325144
Dodecanedioate[b]	Fatty acid, dicarboxylate	HMDB0000623	-0.16	<0.001	<0.01	30318341 and 26085512
1-oleoylglycero[b]	Monoacylglycerol	HMDB0011567	0.14	<0.01	0.02	30318341
Cortisol	Corticosteroids	HMDB0000063	-0.14	<0.01	0.02	30318341 and 35673462
Docosahexaenoate	Long chain polyunsaturated fatty acid (n3 and n6)	HMDB0002183	-0.13	<0.01	0.03	30318341
Carnitine	Carnitine metabolism	HMDB0000062	0.13	<0.01	0.03	30318341 and 26085512
Peptide						
Gamma-glutamylglutamine[b]	Gamma-glutamyl amino acid	HMDB0011738	-0.22	<0.001	<0.001	30318341 and 29325144
Gamma-glutamylvaline[b]	Gamma-glutamyl amino acid	HMDB0011172	0.22	<0.001	<0.001	30318341 and 29325144
Gamma-glutamylphenylalanine[b]	Gamma-glutamyl amino acid	HMDB0000594	0.18	<0.001	<0.01	30318341 and 29325144
Gamma-glutamylleucine[b]	Gamma-glutamyl amino acid	HMDB0011171	0.18	<0.001	<0.01	30318341
Gamma-glutamyltyrosine[b]	Gamma-glutamyl amino acid	HMDB0011741	0.17	<0.001	<0.01	30318341 and 29325144
Prolylhydroxyproline[b]	Urea cycle, arginine and proline metabolism	HMDB0006695	-0.20	<0.001	<0.001	NA
Adsgegdfxaegggvr[b]	Fibrinogen cleavage peptide	NA	0.14	<0.01	0.02	NA
Carbohydrate						
Mannose[b]	Fructose, mannose and galactose metabolism	HMDB0000169	0.22	<0.001	<0.001	30318341
Fructose	Fructose, mannose and galactose metabolism	HMDB0000660	0.15	<0.01	<0.01	30318341
1,5-anhydroglucitol (1,5-AG)[b]	Glycolysis, gluconeogenesis, and pyruvate metabolism	HMDB0002712	-0.15	<0.01	<0.01	30318341
Glycerate	Glycolysis, gluconeogenesis, and pyruvate metabolism	HMDB0000139	-0.12	0.01	0.05	30318341
Arabitol	Pentose metabolism	HMDB0001851	-0.13	<0.01	0.04	30318341

Cofactors and vitamins						
Gamma-tocopherol[b]	Tocopherol metabolism	HMDB0006335	0.24	<0.001	<0.001	30318341 and 29325144
Threonate[b]	Ascorbate and aldarate metabolism	HMDB0062620	-0.19	<0.001	<0.001	30318341 and 29325144
Scyllo-inositol[b]	Cyclohexanols	HMDB0000160	-0.16	<0.001	<0.01	29325144
Heme[b]	Porphyrin metabolism	HMDB03178	-0.14	<0.01	0.01	NA
Xenobiotics						
Piperine[b]	Food component/Plant	HMDB0029377	0.22	<0.001	<0.001	30318341
Quinate[b]	Food component/Plant	HMDB0003072	-0.19	<0.001	<0.01	NA
Catechol sulfate[b]	Benzoate metabolism	HMDB0059724	-0.18	<0.001	<0.01	NA
Benzoate[b]	Benzoate metabolism	HMDB0001870	-0.17	<0.001	<0.01	23862058
Nucleotide						
Urate[b]	Purine metabolism, (Hypo)Xanthine/Inosine containing	HMDB0000289	0.26	<0.001	<0.001	30318341 and 29325144
Energy						
Phosphate[b]	Oxidative phosphorylation	HMDB0001429	-0.16	<0.001	<0.01	21528821

[a] Spearman correlation coefficients were adjusted for age, sex, smoking status (never, former, current), and pack-years of smoking.

[b] The 53 metabolites that were also correlated with weight change from 20 years old to baseline (*p* FDR <0.05).

Abbreviations: PLCO, Prostate, Lung, Colorectal, and Ovarian Cancer Screening Trial; NA, not available; FDR, false discovery rate.

Table S2. The coefficients from the LASSO and Ridge regression models

Metabolite	HMDB	LASSO regression coefficient	Ridge regression coefficient
Amino Acid			
Glutamate	HMDB0000148	0.920415609	0.516161
Asparagine	HMDB0000168	-0.373030474	-0.2798661
Histidine	HMDB0000177	-0.13460453	-0.2868985
Serotonin (5-HT)	HMDB00259	-0.433762706	NA
N-acetylglycine	HMDB0000532	-0.18138198	-0.2462403
Tyrosine	HMDB0000158	0.038744849	0.2032281
Betaine	HMDB0000043	-0.081337773	-0.1919704
Kynurenine	HMDB0000684	0.004934039	0.1686917
Glutamine	HMDB0000641	-0.000539873	-0.2003349
C-glycosyltryptophan	HMDB0240296	0.321668972	0.2098366
N-acetylalanine	HMDB0000766	0.13081444	0.2144665
Creatine	HMDB0000064	0.161833366	0.2028269
Lipid			
Andro steroid monosulfate 2	NA	0.227524563	0.3000439
2-ethylhexanoate	HMDB0062546	-0.019977624	NA
Butyrylcarnitine	HMDB0002013	0.239947416	NA
Palmitoleoyl sphingomyelin	HMDB0010169	-0.409900745	-0.3659114
Epiandrosterone sulfate	HMDB0062657	-0.22664085	-0.1739007
LysoPE(16:0/0:0)	HMDB11503	-0.201251935	NA
Androsterone sulfate	HMDB0002759	-0.033272227	-0.1622673
Cortisol	HMDB0000063	-0.289800913	-0.3022905
LysoPE(0:0/16:0)	HMDB0011473	-0.252697188	NA
Docosahexaenoate	HMDB0002183	-0.206768782	-0.238051
Carnitine	HMDB0000062	0.047234472	0.1608771
Deoxycholate	HMDB0246256	0.007759794	NA
7-HOCA	HMDB0012458	0.359546627	0.2869461
LysoPE(18:0/0:0)	HMDB0011130	-0.374069059	NA
Peptide			
Gamma-glutamylglutamine	HMDB0011738	-0.215507148	NA
Prolylhydroxyproline	HMDB0006695	-0.300133784	NA
Adsgegdfxaegggvr	NA	0.24791978	0.2345603
Carbohydrate			
Mannose	HMDB0000169	0.283501057	0.3015167
Arabitol	HMDB0001851	-0.147683256	-0.2537887
Cofactors and vitamins			
Gamma_tocopherol	HMDB0006335	0.408906207	0.3854707
Scyllo-inositol	HMDB0000160	-0.020473208	NA
Heme	HMDB03178	-0.29154954	NA
Xenobiotics			
Piperine	HMDB0029377	0.351999278	NA
Catechol sulfate	HMDB0059724	-0.396812463	-0.3364843

Benzoate	HMDB0001870	-0.010408274	-0.1944526
Nucleotide			
Urate	HMDB0000289	0.734723773	0.4359744
Energy			
Phosphate	HMDB0001429	-0.214415097	-0.3367624

Abbreviations: LASSO, Least Absolute Shrinkage and Selection Operator; PLCO, Prostate, Lung, Colorectal, and Ovarian Cancer Screening Trial; NA, not available.

Table S3. The 59 metabolites correlated with weight change from 20 years old to baseline among 446 participants from the PLCO cohort[a]

Metabolite	Sub pathway	HMDB	*r*	*p*	*p* FDR
Amino acid					
Betaine	Glycine, serine and threonine metabolism	HMDB0000043	-0.23	<0.001	<0.001
Glycine	Glycine, serine and threonine metabolism	HMDB0000123	-0.22	<0.001	<0.001
N-acetylglycine	Glycine, serine and threonine metabolism	HMDB0000532	-0.20	<0.001	<0.001
Serine	Glycine, serine and threonine metabolism	HMDB0000187	-0.16	<0.01	<0.01
Isoleucine	Leucine, isoleucine and valine metabolism	HMDB0000172	0.15	<0.01	0.01
3-hydroxyisobutyrate	Leucine, isoleucine and valine metabolism	HMDB0000023	0.13	<0.01	0.03
3-methyl-2-oxobutyrate	Leucine, isoleucine and valine metabolism	HMDB0000019	0.15	<0.01	<0.01
Isovalerylcarnitine	Leucine, isoleucine and valine metabolism	HMDB0000688	0.19	<0.001	<0.01
Glutamate	Glutamate metabolism	HMDB0000148	0.33	<0.001	<0.001
Glutamine	Glutamate metabolism	HMDB0000641	-0.14	<0.01	0.03
Kynurenine	Tryptophan metabolism	HMDB0000684	0.13	<0.01	0.04
Serotonin (5-HT)	Tryptophan metabolism	HMDB00259	-0.20	<0.001	<0.001
Asparagine	Alanine and aspartate metabolism	HMDB0000168	-0.24	<0.001	<0.001
Tyrosine	Tyrosine metabolism	HMDB0000158	0.13	<0.01	0.03
Creatine	Creatine metabolism	HMDB0000064	0.15	<0.01	0.01
Histidine	Histidine metabolism	HMDB0000177	-0.20	<0.001	<0.001
Lipid					
LysoPC(18:2(9Z,12Z)/0:0)	Glycerophospholipid	HMDB0010386	-0.20	<0.001	<0.001
2-oleoylglycerophosphocholine	Glycerophospholipid	NA	-0.18	<0.001	<0.01
LysoPC(22:6(4Z,7Z,10Z,13Z,16Z,19Z)/0:0)	Glycerophospholipid	HMDB10404	-0.18	<0.001	<0.01
LysoPC(20:2(11Z,14Z)/0:0)	Glycerophospholipid	HMDB10392	-0.18	<0.001	<0.01
2-linoleoylglycerophosphocholine	Glycerophospholipid	NA	-0.16	<0.001	<0.01
1-heptadecanoylglycerophosphocholine	Glycerophospholipid	NA	-0.16	<0.001	<0.01
LysoPE(16:0/0:0)	Glycerophospholipid	HMDB11503	-0.16	<0.01	<0.01
LysoPC(18:1(9Z)/0:0)	Glycerophospholipid	HMDB02815	-0.17	<0.01	<0.01
Propionylcarnitine	Fatty acid metabolism (also BCAA metabolism)	HMDB0000824	0.18	<0.001	<0.01
Butyrylcarnitine	Fatty acid metabolism (also BCAA metabolism)	HMDB0002013	0.19	<0.001	<0.001
2-methylbutyroylcarnitine	Fatty acid metabolism	HMDB0000378	0.12	<0.01	0.04
2-ethylhexanoate	Medium chain fatty acid	HMDB0062546	-0.17	<0.01	<0.01

Nonadecanoate (19:0)	Long chain saturated fatty acid	HMDB0000772	-0.13	<0.01	0.03
Dodecanedioate	Fatty acid, dicarboxylate	HMDB0000623	-0.15	<0.01	<0.01
Epiandrosterone sulfate	Androgenic steroids	HMDB0062657	-0.15	<0.01	0.01
Andro steroid monosulfate 2	Androgenic steroids	NA	0.24	<0.001	<0.001
4-androsten-3beta,17beta-diol disulfate 1	Androgenic steroids	NA	0.13	<0.01	0.04
4-androsten-3beta,17beta-diol disulfate 2	Androgenic steroids	NA	0.13	<0.01	0.04
1-oleoylglycero	Monoacylglycerol	HMDB0011567	0.16	<0.01	<0.01
2-hydroxybutyrate (AHB)	Glutathione metabolism	HMDB0000008	0.19	<0.001	<0.001
7-HOCA	Bile acids, alcohols and derivativesl	HMDB0012458	0.13	<0.01	0.03
Palmitoyl sphingomyelin	Sphingomyelins	HMDB0010169	-0.19	<0.001	<0.01
Peptide					
Gamma-glutamylglutamine	Gamma-glutamyl amino acid	HMDB0011738	-0.22	<0.001	<0.001
Gamma-glutamylvaline	Gamma-glutamyl amino acid	HMDB0011172	0.21	<0.001	<0.001
Gamma-glutamyltyrosine	Gamma-glutamyl amino acid	HMDB0011741	0.15	<0.01	<0.01
Gamma-glutamylleucine	Gamma-glutamyl amino acid	HMDB0011171	0.15	<0.01	<0.01
Gamma-glutamylphenylalanine	Gamma-glutamyl amino acid	HMDB0000594	0.15	<0.01	<0.01
Prolylhydroxyproline	Urea cycle; arginine and proline metabolism	HMDB0006695	-0.20	<0.001	<0.001
Adsgegdfxaegggvr	Fibrinogen cleavage peptide	NA	0.12	<0.01	0.04
Carbohydrate					
Mannose	Fructose, mannose and galactose metabolism	HMDB0000169	0.21	<0.001	<0.001
1,5-anhydroglucitol (1,5-AG)	Glycolysis, gluconeogenesis, and pyruvate metabolism	HMDB0002712	-0.14	<0.01	0.02
Cofactors and vitamins					
Gamma-tocopherol	Tocopherol metabolism	HMDB0006335	0.24	<0.001	<0.001
Scyllo-inositol	Cyclohexanols	HMDB0000160	-0.16	<0.01	<0.01
Threonate	Ascorbate and aldarate metabolism	HMDB0062620	-0.19	<0.001	<0.01
Heme	Porphyrin metabolism	HMDB03178	-0.15	<0.01	0.01
Xenobiotics					
Catechol sulfate	Benzoate metabolism	HMDB0059724	-0.18	<0.001	<0.01
Benzoate	Benzoate metabolism	HMDB0001870	-0.15	<0.01	0.01
Hippurate	Benzoate metabolism	HMDB0062583	-0.17	<0.001	<0.01
Piperine	Food component/Plant	HMDB0029377	0.22	<0.001	<0.001
Quinate	Food component/Plant	HMDB0003072	-0.17	<0.001	<0.01

Nucleotide					
Urate	Purine metabolism, (hypo)xanthine/Inosine containing	HMDB0000289	0.24	<0.001	<0.001
Pseudouridine	Pyrimidine metabolism, uracil containing	HMDB0000767	0.13	<0.01	0.04
Energy					
Phosphate	Oxidative phosphorylation	HMDB0001429	-0.16	<0.001	<0.01

[a] Spearman correlation coefficients were adjusted for age, sex, smoking status (never, former, current), and pack-years of smoking.

Abbreviations: PLCO, Prostate, Lung, Colorectal, and Ovarian Cancer Screening Trial; NA, not available; FDR, false discovery rate.

Table S4. Associations between BMI-related metabolites and colorectal cancer risk in the PLCO and Jiangsu cohorts

Cohort	Metabolites	Quartiles of metabolites, OR (95% CI)[a]				*p* for trend	OR per 1-SD increase
		Q1	Q2	Q3	Q4		
PLCO	Glutamine	1 (referent)	0.80 (0.45-1.42)	0.74 (0.41-1.36)	0.58 (0.30-1.10)	0.01	0.72 (0.57-0.92)
	Histidine	1 (referent)	0.73 (0.40-1.34)	0.84 (0.46-1.54)	0.41 (0.21-0.80)	0.01	0.73 (0.57-0.92)
	Gamma-glutamylglutamine	1 (referent)	0.82 (0.46-1.47)	0.82 (0.44-1.54)	0.49 (0.25-0.96)	0.01	0.72 (0.56-0.93)
	Andro steroid monosulfate 2	1 (referent)	1.39 (0.73-2.67)	1.48 (0.75-2.91)	1.79 (0.91-3.53)	0.04	1.27 (1.01-1.61)
Jiangsu	Glutamine	1 (referent)	0.56 (0.26-1.20)	0.87 (0.26-2.90)	0.49 (0.13-1.78)	0.02	0.50 (0.28-0.90)
	Histidine	1 (referent)	0.61 (0.32-1.19)	0.62 (0.27-1.42)	0.68 (0.29-1.58)	0.57	0.92 (0.69-1.23)
	Andro steroid monosulfate 2	1 (referent)	1.10 (0.60-2.03)	1.45 (0.73-2.89)	1.31 (0.61-2.84)	0.24	1.18 (0.89-1.56)

[a] Conditional logistic regression model adjusted for age, smoking status, pack-years of smoking, alcohol intake, diabetes history, and in the PLCO, study center and family history of colorectal cancer.
Abbreviations: PLCO, Prostate, Lung, Colorectal, and Ovarian Cancer Screening Trial; OR, odds ratio; CI, confidence interval; SD, standard deviation.
